# Supplementary material for: Altered N-glycan composition impacts flagella-mediated adhesion in Chlamydomonas reinhardtii
Source: eLife. 2020 Dec 10;9:e58805. doi: 10.7554/eLife.58805 (PMC7759384; doi:10.7554/eLife.58805)
Supplement: Figure 4—source data 1. [file elife-58805-fig4-data1.docx]

| **Strains**  **Adhesion Force**  **(nN)** | **WT- SAG**  **+ Ciliobrevin D** | **WT- SAG**  **- Ciliobrevin D** | **CRISPR*_XylT1A_***  **+ Ciliobrevin D** | **CRISPR*_XylT1A_***  **- Ciliobrevin D** |
| --- | --- | --- | --- | --- |
| **minimum** | 1.02 | 0.59 | 1.07 | 0.38 |
| **25^th^ percentile** | 2.08 | 1.02 | 1.44 | 0.70 |
| **median** | 2.63 | 1.50 | 1.84 | 1.08 |
| **75^th^ percentile** | 3.49 | 2.18 | 2.13 | 1.78 |
| **maximum** | 4.45 | 3.63 | 3.57 | 2.75 |

**Figure 4 – Source Data 1: Adhesion force under blue light**

| **Strains**  **Adhesion Force**  **(nN)** | **WT- SAG**  **+ Ciliobrevin D** | **WT- SAG**  **- Ciliobrevin D** | **CRISPR*_XylT1A_***  **+ Ciliobrevin D** | **CRISPR*_XylT1A_***  **- Ciliobrevin D** |
| --- | --- | --- | --- | --- |
| **minimum** | 1.02 | 0.59 | 1.07 | 0.38 |
| **25^th^ percentile** | 2.08 | 1.02 | 1.44 | 0.70 |
| **median** | 2.63 | 1.50 | 1.84 | 1.08 |
| **75^th^ percentile** | 3.49 | 2.18 | 2.13 | 1.78 |
| **maximum** | 4.45 | 3.63 | 3.57 | 2.75 |

**Figure 4 – Source Data 2: Adhesion force under red light**

| **Strains**  **Adhesion Force**  **(nN)** | **WT- SAG**  **+ Ciliobrevin D** | **WT- SAG**  **- Ciliobrevin D** | **CRISPR*_XylT1A_***  **+ Ciliobrevin D** | **CRISPR*_XylT1A_***  **- Ciliobrevin D** |
| --- | --- | --- | --- | --- |
| **minimum** | 0.04 | 0.03 | 0.02 | 0.06 |
| **25^th^ percentile** | 0.07 | 0.08 | 0.05 | 0.11 |
| **median** | 0.10 | 0.13 | 0.09 | 0.21 |
| **75^th^ percentile** | 0.19 | 0.18 | 0.30 | 0.30 |
| **maximum** | 0.35 | 0.27 | 0.41 | 0.42 |

**Figure 4 – Source Data 3: Adhesion force of each cell under blue light**

| **WT- SAG**  **+ Ciliobrevin D**  **(N=23cells)** | **WT- SAG**  **- Ciliobrevin D**  **(N=32cells)** | **CRISPR*_XylT1A_***  **+ Ciliobrevin D**  **(N=24cells)** | **CRISPR*_XylT1A_***  **- Ciliobrevin D**  **(N=28cells)** |
| --- | --- | --- | --- |
| \| 1.02 \| \| --- \| \| 1.15 \| \| 1.69 \| \| 1.82 \| \| 2.02 \| \| 2.08 \| \| 2.09 \| \| 2.12 \| \| 2.51 \| \| 2.52 \| \| 2.53 \| \| 2.63 \| \| 2.70 \| \| 2.74 \| \| 2.82 \| \| 3.02 \| \| 3.22 \| \| 3.59 \| \| 3.61 \| \| 3.61 \| \| 3.67 \| \| 4.45 \| \| 5.62 \| | \| 0.59 \| \| --- \| \| 0.62 \| \| 0.70 \| \| 0.73 \| \| 0.93 \| \| 0.95 \| \| 0.98 \| \| 0.99 \| \| 1.05 \| \| 1.07 \| \| 1.08 \| \| 1.19 \| \| 1.30 \| \| 1.31 \| \| 1.34 \| \| 1.49 \| \| 1.51 \| \| 1.71 \| \| 1.75 \| \| 1.76 \| \| 1.81 \| \| 1.94 \| \| 1.97 \| \| 2.10 \| \| 2.26 \| \| 2.49 \| \| 2.54 \| \| 2.65 \| \| 2.66 \| \| 3.63 \| \| 4.16 \| \| 4.19 \| | \| 1.07 \| \| --- \| \| 1.21 \| \| 1.37 \| \| 1.39 \| \| 1.41 \| \| 1.42 \| \| 1.46 \| \| 1.49 \| \| 1.51 \| \| 1.74 \| \| 1.75 \| \| 1.83 \| \| 1.85 \| \| 1.87 \| \| 1.91 \| \| 2.02 \| \| 2.05 \| \| 2.12 \| \| 2.14 \| \| 2.25 \| \| 2.47 \| \| 2.93 \| \| 3.06 \| \| 3.40 \| | \| 0.38 \| \| --- \| \| 0.39 \| \| 0.55 \| \| 0.56 \| \| 0.56 \| \| 0.60 \| \| 0.68 \| \| 0.73 \| \| 0.74 \| \| 0.75 \| \| 0.76 \| \| 0.80 \| \| 0.89 \| \| 0.95 \| \| 1.21 \| \| 1.33 \| \| 1.35 \| \| 1.41 \| \| 1.44 \| \| 1.70 \| \| 1.77 \| \| 1.80 \| \| 1.86 \| \| 1.92 \| \| 1.92 \| \| 2.05 \| \| 2.52 \| \| 2.75 \| |

**Figure 4 – Source Data 4: Adhesion force of each cell under red light**

| **WT- SAG**  **+ Ciliobrevin D**  **(N=23cells)** | **WT- SAG**  **- Ciliobrevin D**  **(N=32cells)** | **CRISPR*_XylT1A_***  **+ Ciliobrevin D**  **(N=24cells)** | **CRISPR*_XylT1A_***  **- Ciliobrevin D**  **(N=28cells)** |
| --- | --- | --- | --- |
| \| \| 0.04 \| \| --- \| \| 0.04 \| \| 0.04 \| \| 0.05 \| \| 0.05 \| \| 0.07 \| \| 0.07 \| \| 0.07 \| \| 0.08 \| \| 0.08 \| \| 0.08 \| \| 0.10 \| \| 0.11 \| \| 0.14 \| \| 0.14 \| \| 0.16 \| \| 0.18 \| \| 0.19 \| \| 0.22 \| \| 0.28 \| \| 0.30 \| \| 0.35 \| \| 0.42 \| \| \| --- \| --- \| --- \| --- \| --- \| --- \| --- \| --- \| --- \| --- \| --- \| --- \| --- \| --- \| --- \| --- \| --- \| --- \| --- \| --- \| --- \| --- \| --- \| --- \| | \| 0.03 \| \| --- \| \| 0.05 \| \| 0.05 \| \| 0.06 \| \| 0.06 \| \| 0.07 \| \| 0.07 \| \| 0.08 \| \| 0.09 \| \| 0.09 \| \| 0.09 \| \| 0.09 \| \| 0.10 \| \| 0.11 \| \| 0.11 \| \| 0.13 \| \| 0.13 \| \| 0.13 \| \| 0.14 \| \| 0.14 \| \| 0.14 \| \| 0.16 \| \| 0.17 \| \| 0.17 \| \| 0.18 \| \| 0.19 \| \| 0.21 \| \| 0.21 \| \| 0.23 \| \| 0.25 \| \| 0.27 \| \| 0.34 \| | \| \| 0.02 \| \| --- \| \| 0.02 \| \| 0.03 \| \| 0.04 \| \| 0.04 \| \| 0.05 \| \| 0.05 \| \| 0.05 \| \| 0.06 \| \| 0.06 \| \| 0.09 \| \| 0.09 \| \| 0.10 \| \| 0.12 \| \| 0.12 \| \| 0.16 \| \| 0.23 \| \| 0.26 \| \| 0.33 \| \| 0.34 \| \| 0.35 \| \| 0.41 \| \| 2.25 \| \| 3.40 \| \| \| --- \| --- \| --- \| --- \| --- \| --- \| --- \| --- \| --- \| --- \| --- \| --- \| --- \| --- \| --- \| --- \| --- \| --- \| --- \| --- \| --- \| --- \| --- \| --- \| --- \| \|  \| \|  \| \|  \| \|  \| \|  \| \|  \| \|  \| \|  \| \|  \| \|  \| \|  \| \|  \| \|  \| \|  \| \|  \| \|  \| \|  \| \|  \| \|  \| \|  \| \|  \| \|  \| \|  \| | \| \| 0.06 \| \| --- \| \| 0.09 \| \| 0.09 \| \| 0.09 \| \| 0.09 \| \| 0.10 \| \| 0.11 \| \| 0.12 \| \| 0.12 \| \| 0.14 \| \| 0.16 \| \| 0.19 \| \| 0.19 \| \| 0.20 \| \| 0.21 \| \| 0.22 \| \| 0.22 \| \| 0.23 \| \| 0.25 \| \| 0.29 \| \| 0.30 \| \| 0.31 \| \| 0.34 \| \| 0.34 \| \| 0.36 \| \| 0.39 \| \| 0.42 \| \| 0.61 \| \| \| --- \| --- \| --- \| --- \| --- \| --- \| --- \| --- \| --- \| --- \| --- \| --- \| --- \| --- \| --- \| --- \| --- \| --- \| --- \| --- \| --- \| --- \| --- \| --- \| --- \| --- \| --- \| --- \| --- \| \|  \| \|  \| \|  \| \|  \| \|  \| \|  \| \|  \| \|  \| \|  \| \|  \| \|  \| \|  \| \|  \| \|  \| \|  \| \|  \| \|  \| \|  \| \|  \| \|  \| \|  \| \|  \| \|  \| \|  \| \|  \| \|  \| \|  \| |
