# Supplementary material for: Altered N-glycan composition impacts flagella-mediated adhesion in Chlamydomonas reinhardtii
Source: eLife. 2020 Dec 10;9:e58805. doi: 10.7554/eLife.58805 (PMC7759384; doi:10.7554/eLife.58805)
Supplement: Transparent reporting form [file elife-58805-transrepform.docx]

***eLife’s* transparent reporting form**

We encourage authors to provide detailed information *within their submission* to facilitate the interpretation and replication of experiments. Authors can upload supporting documentation to indicate the use of appropriate reporting guidelines for health-related research (see [EQUATOR Network](http://www.equator-network.org/%20)), life science research (see the [BioSharing Information Resource](https://biosharing.org/)), or the [ARRIVE guidelines](http://www.plosbiology.org/article/info:doi/10.1371/journal.pbio.1000412) for reporting work involving animal research. Where applicable, authors should refer to any relevant reporting standards documents in this form.

If you have any questions, please consult our Journal Policies and/or contact us: [editorial@elifesciences.org](mailto:editorial@elifesciences.org).

**Sample-size estimation**

- You should state whether an appropriate sample size was computed when the study was being designed
- You should state the statistical method of sample size computation and any required assumptions
- If no explicit power analysis was used, you should describe how you decided what sample (replicate) size (number) to use

Please outline where this information can be found within the submission (e.g., sections or figure legends), or explain why this information doesn’t apply to your submission:

No explicit power analysis was used.

**Replicates**

- You should report how often each experiment was performed
- You should include a definition of biological versus technical replication
- The data obtained should be provided and sufficient information should be provided to indicate the number of independent biological and/or technical replicates
- If you encountered any outliers, you should describe how these were handled
- Criteria for exclusion/inclusion of data should be clearly stated
- High-throughput sequence data should be uploaded before submission, with a private link for reviewers provided (these are available from both GEO and ArrayExpress)

Please outline where this information can be found within the submission (e.g., sections or figure legends), or explain why this information doesn’t apply to your submission:

**Flagella length and flagellation:** Three independent biological replicates were performed with minimum 100 cells per replicates analyzed. Information can be obtained from material and methods.

**Protein abundances:** Protein abundance rations were calculated based on three independent biological replicates.

**Microbead measurements:** Three biological replicates were performed with 50 cells analyzed per replicate. Information can be obtained from figure legend of figure 2.

**AFM Measurements:** Three biological replicates were performed with minimum 5 cells per replicate analyzed. Information can be found in figure legend of Figure 3.

**Micropipette force measurements:** N of micropipette force measurements can be obtained from Figure 4. 10 force measurements were performed per cell analyzed.

**IFT- and Gliding-velocities:** Three biological replicates were performed with 10 cells in gliding configuration per replicate analyzed. Information can be found in figure legend of Figure 5.

**Statistical reporting**

- Statistical analysis methods should be described and justified
- Raw data should be presented in figures whenever informative to do so (typically when N per group is less than 10)
- For each experiment, you should identify the statistical tests used, exact values of N, definitions of center, methods of multiple test correction, and dispersion and precision measures (e.g., mean, median, SD, SEM, confidence intervals; and, for the major substantive results, a measure of effect size (e.g., Pearson's r, Cohen's d)
- Report exact p-values wherever possible alongside the summary statistics and 95% confidence intervals. These should be reported for all key questions and not only when the p-value is less than 0.05.

Please outline where this information can be found within the submission (e.g., sections or figure legends), or explain why this information doesn’t apply to your submission:

**Figure 2:** Data shown represents mean of three replicates à 50 cells. Error bars show SEM. T-test was performed to compare variation from mean between WT and mutants.

**Figure 3:** Two sided, two sample t-test was performed to compare variation from mean between WT and mutants.

**Figure 4:** Statistical analysis was performed via Mann-Whitney U test on mean value of 10 measurements per cell.

**Figure 5: A,** Two sided, two sample t-test was performed to compare variation from mean between WT and mutants. **C,** T-test was performed on mean values of three replicates in regard of proportion of n-gliding events and IFT velocities. Distribution of gliding velocities was analyzed via Mann-Whitney U test.

(For large datasets, or papers with a very large number of statistical tests, you may upload a single table file with tests, Ns, etc., with reference to sections in the manuscript.)

**Group allocation**

- Indicate how samples were allocated into experimental groups (in the case of clinical studies, please specify allocation to treatment method); if randomization was used, please also state if restricted randomization was applied
- Indicate if masking was used during group allocation, data collection and/or data analysis

Please outline where this information can be found within the submission (e.g., sections or figure legends), or explain why this information doesn’t apply to your submission:

No experimental groups were used in this study.

**Additional data files (“source data”)**

- We encourage you to upload relevant additional data files, such as numerical data that are represented as a graph in a figure, or as a summary table
- Where provided, these should be in the most useful format, and they can be uploaded as “Source data” files linked to a main figure or table
- Include model definition files including the full list of parameters used
- Include code used for data analysis (e.g., R, MatLab)
- Avoid stating that data files are “available upon request”

Please indicate the figures or tables for which source data files have been provided:

The mass spectrometry proteomics data (Figure 1) have been deposited to the ProteomeXchange Consortium ([http://proteomecentral.proteomexchange.org](http://proteomecentral.proteomexchange.org/)) via the PRIDE partner repository with the dataset identifier PXD018353 and will be publically available upon acceptance of the manuscript. During peer review, the dataset can be entered via the account reviewer44250@ebi.ac.uk and the password OLy6xQJY. For further requests, please contact K. Huang (huangky@ihb.ac.cn) or M. Hippler (mhippler@uni-muenster.de).
